# A New Color-reaction (Rossbach’s) of the Urine

**Published:** 1889-03

**Authors:** 


A NEW COLOR-REACTION (ROSSBACH'S) OF THE URINE.—Some urines will, upon the addition of nitric acid to the boiling urine, change their color to a red, burgundy-red and bluish-red with bluish-red foam. Further addition of nitric acid, drop by drop, will suddenly change the color into reddish-yellow and yellow, the foam being likewise yellow. Neutralizing slowly with ammonia, after each drop bluish-red deposits occur, which dissolve again till finally a reddish-brown color remains constant. The clinical importance of the reaction, which seems to depend upon the indolphenol components in the urine, is decided, inasmuch it always appears in earnest intestinal derangements—occlusion, or cancer of the intestine, extensive ulcerations, and severe diarrheas. Peritonitis and diseases of the stomach, paratyphlitis, do not cause the reaction to appear. The longer persistence of the reaction has always a grave outlook.—*Berlin. klin. Wochenschr*, Jan. 7, 1889; page 5.

